# A Mathematical Model of a Novel 3D Fractal-Inspired Piezoelectric Ultrasonic Transducer

**DOI:** 10.3390/s16122170

**Published:** 2016-12-17

**Authors:** Sara Canning, Alan J. Walker, Paul A. Roach

**Affiliations:** 1 School of Computing and Mathematics, University of South Wales, Pontypridd CF37 1DL, UK; sara.koubayssi@southwales.ac.uk (S.C.); paul.roach@southwales.ac.uk (P.A.R.); 2 School of Science and Sport, University of the West of Scotland, Paisley PA1 2BE, UK

**Keywords:** piezoelectric materials, mathematical modelling, Sierpinski tetrix fractal, ultrasonic transducer, renormalization, finite differences

## Abstract

Piezoelectric ultrasonic transducers have the potential to operate as both a sensor and as an actuator of ultrasonic waves. Currently, manufactured transducers operate effectively over narrow bandwidths as a result of their regular structures which incorporate a single length scale. To increase the operational bandwidth of these devices, consideration has been given in the literature to the implementation of designs which contain a range of length scales. In this paper, a mathematical model of a novel Sierpinski tetrix fractal-inspired transducer for sensor applications is presented. To accompany the growing body of research based on fractal-inspired transducers, this paper offers the first sensor design based on a three-dimensional fractal. The three-dimensional model reduces to an effective one-dimensional model by allowing for a number of assumptions of the propagating wave in the fractal lattice. The reception sensitivity of the sensor is investigated. Comparisons of reception force response (RFR) are performed between this novel design along with a previously investigated Sierpinski gasket-inspired device and standard Euclidean design. The results indicate that the proposed device surpasses traditional design sensors.

## 1. Introduction

Ultrasonic transducers have the ability to generate and detect ultrasonic waves. Ultrasonic sensors, that is, ultrasonic transducers operating in reception mode, convert mechanical energy into electrical energy through the contact of sound waves resulting in the production of an electrical signal [1,2,3]. Ultrasonic sensors are used extensively in fields such as communication, medicine and non-destructive evaluation [4,5]. The regular geometry of traditional ultrasonic sensors restricts their effective performance to a small range of frequencies, due to their one dominant length scale. In contrast, biological sensors found in natural systems have greater complexity in their design, including a range of length scales [6,7,8]. This results in far more effective reception of ultrasonic waves over a wider range of frequencies. Construction of devices which benefit from a range of length scales, similar to those found in nature, is therefore of great interest.

Fractals are complex geometrical objects which exhibit structural similarity across magnification levels in addition to being constructed from a range of length scales. To assist the construction of devices that operate efficiently over a larger range of frequencies, it is possible to consider the implementation of fractal-like structures into the design of new ultrasonic transducers [6,9,10,11]. Research into fractal-inspired transducers has included the plane wave expansion model to study the performances of a Sierpinski carpet and Cantor set pre-fractal transducers [9]. The Green function renormalization method was utilised to obtain analytical results for a Sierpinski gasket-inspired transducer [6] and was further investigated using a finite element approach [11]. A prototype of its fourth fractal generation level was manufactured [10]. It was shown in these papers that ultrasonic transducers with multiple length scales benefited from more resonances and improved operation over a larger range of frequencies.

In this paper, the Green function renormalization method is utilised to analyse the potential performance of a three-dimensional fractal-inspired ultrasound sensor. An expression for the reception force response (RFR) is derived and plotted against the operating frequency. This profile is then examined for the purpose of comparing against the standard (Euclidean) and another previously investigated fractal-inspired design. The results are indicative of an improvement in the RFR bandwidth in comparison to the Euclidean transducer and comparable to the previously investigated Sierpinski gasket-inspired transducer.

The investigation of the Sierpinski tetrix fractal-inspired sensor begins in Section 2, where the construction of the Sierpinski tetrix and its lattice counterpart are outlined. In Section 3, the governing wave equation is derived from the general tensor equations. The application of the Green function renormalization method is then utilized in Section 4 to obtain the relevant recursive relations for the fractal-inspired sensor model. In Section 5, the boundary conditions for the model are derived together with an expression for the RFR. In Section 6, the results for the Sierpinski tetrix fractal-inspired sensor are compared with the standard Euclidean design and the Sierpinski gasket fractal-inspired design. The findings are summarised and future research is discussed in Section 7 and Section 8.

## 2. Lattice Structure of the Sierpinski Tetrix

**Figure 1:**
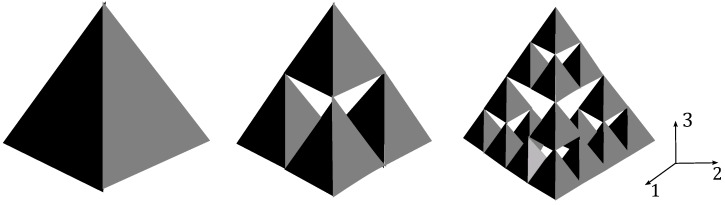
Schematic representation of a Sierpinski tetrix sensor at fractal generation levels one, two and three.

The self-similar structure used in this paper to imitate the complex geometry found in natural ultrasonic sensors is the Sierpinski tetrix. The Sierpinski tetrix can be classified as the three-dimensional equivalent of the Sierpinski gasket on account of its construction [12,13,14]. Thus, its structure is achieved in a similar way whereby the fractal is formed from an initial regular tetrahedron. The following generation is obtained by replacing this initial tetrahedron with four copies of its three-dimensional self. Subsequent generations are then found by repeatedly applying this procedure, and, after an infinite number of iterations, the Sierpinski tetrix fractal is formed. The Sierpinski tetrix pre-fractals at generation levels one, two and three are illustrated in Figure 1.

**Figure 2:**
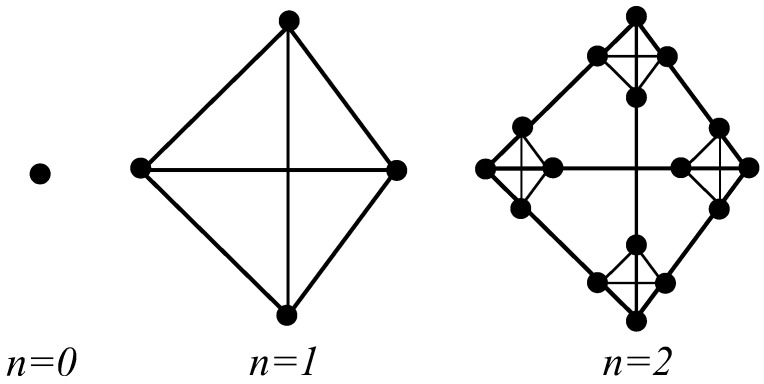
Graphical representations of generations 0 to 2 for the sequence of Sierpinski tetrix lattices.

A lattice counterpart of the Sierpinski tetrix will be used to investigate the propagation of an ultrasonic wave. The lattice represents the vibrations of the piezoelectric material of the Sierpinski tetrix. The interaction between the electrical and mechanical behaviour of the lattice vertices is decribed by [15] and detailed further in Section 3. These lattice structures are constructed by assigning a vertex to the centre of the piezoelectric tetrahedron and joining these vertices by an edge. The process of forming the graph $G^{\left(n+1\right)}$ is obtained by connecting together four copies of the previous graph, $G^{\left(n\right)}$ [6,16,17]; see Figure 2.

For the lattice structure, the *n*th generation graph has $N_{n}=4^{n}$ vertices and the coordination number for this fractal lattice is q=4. However, this coordination number is not consistent since the corner vertices have a coordination number q=3. These corner vertices act as the input and output vertices and interact with external loads. Fictitious vertices *A*, *B*, *C* and *D* are attached to the input/output vertices to accommodate the boundary conditions and to make the coordination number consistent, with q=4. This is analogous to the investigation of the Sierpinski gasket [6]. The length of the fractal lattice, that is the length of the ceramic component of the device (*l*), remains fixed throughout the construction process. Thus, the edge length between adjacent vertices will decrease as the generation level is increased. The recursive relationship of the graphs can be described in terms of their adjacency matrix

(1) $$H^{\left(n+1\right)}={\bar{H}}^{\left(n\right)}+V^{\left(n\right)},$$

where ${\bar{H}}^{\left(n\right)}$ is a block diagonal matrix whose *v* blocks are equal to $H^{\left(n\right)}$, and $V^{\left(n\right)}$ is a sparse matrix that assigns the number one to the connection of sub-graphs and zero otherwise [6,16,18]. The recursion relationship equation is given by [16]

(2) $${\hat{G}}^{\left(n+1\right)}={\bar{G}}^{\left(n\right)}+{\bar{G}}^{\left(n\right)}V^{\left(n\right)}{\hat{G}}^{\left(n+1\right)},$$

where ${\hat{G}}^{\left(n\right)}$ is the Green function matrix which does not account for boundary conditions, and ${\bar{G}}^{\left(n\right)}$ is a block diagonal matrix whose *v* blocks equal ${\hat{G}}^{\left(n\right)}$. To account for boundary conditions, the following equation is used

(3) $$G^{\left(n\right)}={\hat{G}}^{\left(n\right)}+{\hat{G}}^{\left(n\right)}B^{\left(n\right)}G^{\left(n\right)},$$

where $B^{\left(n\right)}$ is a matrix containing the boundary conditions at the input and output vertices.

## 3. Wave Propagation in the Sierpinski Tetrix

The movement of a wave through the piezoelectric material can be described using the stress equation of motion

(4) $$\rho_{T}{\ddot{u}}_{i}=T_{ij,j}\;\;\mathrm{for}\;\;i,j=1,2,3,$$

where $\rho_{T}$ is the density of the piezoelectric material, $u_{i}$ is the displacement tensor and $T_{ij}$ is the stress tensor. The three-dimensional constitutive equations describing the coupling between electrical and mechanical behaviour of the lattice vertices are given by

(5) $$T_{ij}=c_{ijkl}S_{kl}-e_{kij}E_{k},\;\;\;\;D_{i}=e_{ikl}S_{kl}+\epsilon_{ik}E_{k}.$$

In these equations, $c_{ijkl}$ denotes the stiffness tensor, $S_{kl}$ is the strain tensor, $e_{kij}$ is the piezoelectric tensor, $E_{k}$ is the electric field, $D_{i}$ is the displacement and $\epsilon_{ik}$ denotes the permittivity tensor. The relationship between the strain tensor and mechanical displacement is given by

(6) $$S_{kl}=\frac{1}{2}\left(u_{l,k}+u_{k,l}\right).$$

Additionally, a relationship between electric field and electric potential, *ϕ*, is expressed as

(7) $$E_{k}=-\phi_{\;k}.$$

The poling direction of the piezoelectric material is parallel to the direction of the $x_{3}$-axis, and it is assumed that an electric field is only applied in this direction [19,20]. This is to ensure that the orientation of the dipoles of the piezoelectric ceramic are aligned and not left in their randomly oriented state. If left in their original state, the piezoelectric effect is disabled. Previous experimental research on the Sierpinski gasket [10] has shown the dependence of the transducer’s performance on the poling direction (which can be chosen in the manufacturing process). Consequently, the assumption made above on the poling direction and form of the electric field essentially allows for the reduction of the three-dimensional coupling equations in Equation (5) to reduce to one dimension. Hence,

(8) $$E_{1}=E_{2}=0,\;E_{3}\neq 0\;\;\mathrm{and}\;\;D_{1}=D_{2}=0,\;D_{3}\neq 0.$$

Consequently, in Equation (4),

(9) $$T_{ij,j}=T_{3j,j}=T_{33,3}+T_{32,2}+T_{31,1}=T_{3}+T_{4}+T_{5},$$

where the components with i=j relate to longitudinal stresses and with i≠j indicate the shear stresses. Equation (4) can be reduced further since it is assumed that the wave travelling within the sensor is wholly longitudinal along the $x_{3}$-axis, that is, the shear waves are negligible in comparison to that of the longitudinal waves. This assumption can be made due to the poling direction and the direction of the applied electric field and simplifies the algebra considerably. Longitudinal waves in the $x_{1}$- and $x_{2}$-directions are also neglected due to the direction of the poling and the applied electric field. Hence,

(10) $$\rho_{T}{\ddot{u}}_{3}=T_{3}.$$

Electric charges do not flow easily within the piezoelectric ceramic as a result of it being a good insulator. Thus, from Gauss’ law,

(11) $$D_{3,3}=0.$$

Using Equations (5)–(8) and (11) together with Equation (10) yields

(12) $$\rho_{T}{\ddot{u}}_{3}=c_{33}\left(1+\frac{e_{33}^{2}}{\epsilon_{33}c_{33}}\right)\frac{\partial^{2}u_{3}}{\partial x_{3}^{2}}.$$

Equation (12) can be reduced into the one-dimensional form by setting the Young’s modulus of the piezoelectric material, $Y_{T}=c_{33}+e_{33}^{2}/\epsilon_{33}$, and temporarily dropping the subscript attached to the displacement tensor. Thus,

(13) $$\rho_{T}\frac{\partial^{2}u}{\partial t^{2}}=Y_{T}\frac{\partial^{2}u}{\partial x^{2}}.$$

The one-dimensional equation is subsequently used to derive the boundary conditions and RFR. It is discretized and, by introducing the non-dimensionalized variable *θ*, can be written as

(14) $$\frac{\partial^{2}\underline{u}}{\partial \theta^{2}}=A^{\left(n\right)}\underline{u}+B^{\left(n\right)}\underline{u}+{\underline{c}}^{\left(n\right)},$$

where $\theta =\sqrt{(Y_{T}/\rho_{T})t}/\Delta x$, $A^{\left(n\right)}$ represents the discretized Laplacian at fractal generation level *n*, $B^{\left(n\right)}$ and ${\underline{c}}^{\left(n\right)}$ are dictated by the boundary conditions at the input/output vertices, and $\Delta x=l/(2^{n}-1)$ is the length of each edge of the fractal lattice. The size of the ceramic component *l* is fundamental in the sensor design, since the value for *l* will affect the overall performance of the device.

The derivation of the effective one-dimensional model was the technique for simplifying the analysis of wave propagation within the fractal-like lattice. Longitudinal waves can propagate in all three forms, solid, liquid and gases, while shear waves can only propagate through solids [21,22,23]. This effective one-dimensional model accounts only for longitudinal waves in the $x_{3}$-direction since the shear waves and longitudinal waves in the $x_{1}$- and $x_{2}$-directions are considered negligible in comparison. This form of analysis, in effect, treats elastic solids as liquids, simplifying the model while still providing useful results on the likely performance of this sensor. Furthermore, this simplification provides a fair comparison against the previously investigated Sierpinski gasket inspired transducer [6]. Nevertheless, reducing the system to a one-dimensional model will result in the loss of some nonlinear information. This could be resolved in using a fully three-dimensional finite element analysis on the wave propagation. However, while the finite element models proposed [11,24] for the Sierpinski gasket will be more accurate, they are computationally expensive and the finite differences model proposed previously for the same structure [10] produced similar results without the computational overheads. Hence, a similar approach was taken here.

## 4. Renormalization Analysis

In previous research [6,11,25], the Green function renormalization method has been applied to fractal structures with the aim of obtaining recursion relations to facilitate expressions for the transducers’ operating characteristics. In this section, this approach is applied to develop the recursion relations for the Sierpinski tetrix lattice.

It is assumed that Sierpinski tetrix fractal-inspired sensor will have a single input vertex at vertex *A* and three output vertices at vertices *B*, *C* and *D*.

**Figure 3:**
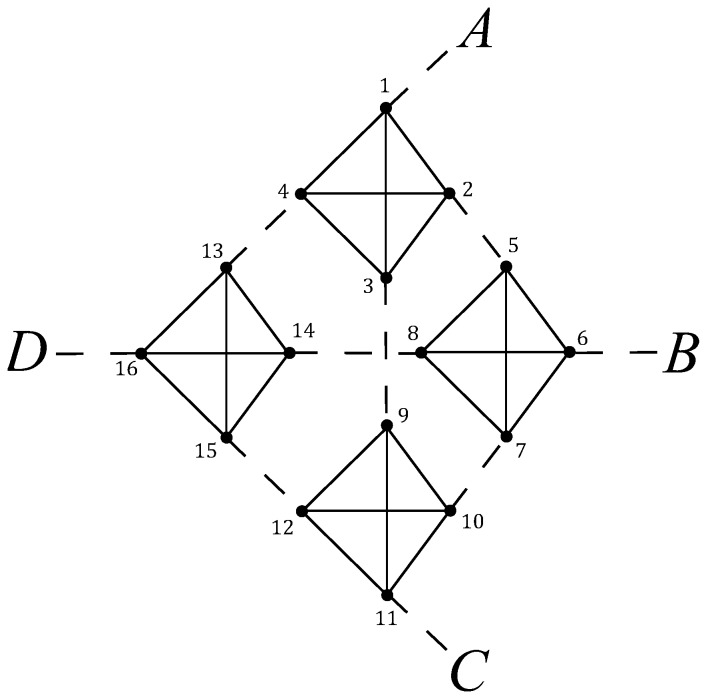
Sierpinski tetrix lattice at generation n=2. Fictitious vertices *A*, *B*, *C* and *D* are introduced to accommodate the boundary conditions.

Figure 3 shows the development of the second generation level fractal lattice from the connection of four copies of the first generation lattice, i.e., the process of forming $G^{\left(n+1\right)}$ is made from the connection of four copies of $G^{\left(n\right)}$. Due to the symmetries of the lattice structure, it is of interest only to obtain the pivotal Green functions. The pivotal Green functions are the minimum number of independent elements required to develop a recursion [16]. While this method can be used at any generation level, the vertex labelling as illustrated in Figure 3, and as mentioned throughout this paper, refers specifially to generation level n=2. The Sierpinski tetrix has two pivotal Green functions, ${\hat{G}}_{1\;1}^{\left(n\right)}$ and ${\hat{G}}_{1\;6}^{\left(n\right)}$, where, for ease of notation, these will be labelled ${\hat{x}}_{t}={\hat{G}}_{1\;1}^{\left(n\right)}$ and ${\hat{y}}_{t}={\hat{G}}_{1\;6}^{\left(n\right)}$. The subscripts attached refer to vertices connecting the lattice structure to the fictitious vertices *A*, *B*, *C* and *D* [6] and the subscript *t* relates to the Sierpinski tetrix model. The corresponding Green functions at the next fractal generation level, ${\hat{X}}_{t}={\hat{G}}_{1\;1}^{\left(n+1\right)}$ and ${\hat{Y}}_{t}={\hat{G}}_{1\;6,}^{\left(n+1\right)}$ are found by applying Equation (2) yielding,

(15) $${\hat{X}}_{t}={\hat{x}}_{t}+3{\hat{y}}_{t}{\hat{G}}_{5\;1}^{\left(n+1\right)},$$

(16) $${\hat{Y}}_{t}={\hat{y}}_{t}\left({\hat{G}}_{2\;1}^{\left(n+1\right)}+{\hat{G}}_{7\;1}^{\left(n+1\right)}\right).$$

To obtain ${\hat{X}}_{t}$ and ${\hat{Y}}_{t}$ in terms of ${\hat{x}}_{t}$ and ${\hat{y}}_{t}$ solely, expressions for ${\hat{G}}_{2\;1,}^{\left(n+1\right)}$ ${\hat{G}}_{5\;1}^{\left(n+1\right)}$ and ${\hat{G}}_{7\;1}^{\left(n+1\right)}$ are required; utilising Equation (2) results in

(17) $${\hat{G}}_{2\;1}^{\left(n+1\right)}={\hat{y}}_{t}+\left({\hat{x}}_{t}+2{\hat{y}}_{t}\right){\hat{G}}_{5\;1}^{\left(n+1\right)},$$

(18) $${\hat{G}}_{5\;1}^{\left(n+1\right)}={\hat{x}}_{t}{\hat{G}}_{2\;1}^{\left(n+1\right)}+2{\hat{y}}_{t}{\hat{G}}_{7\;1}^{\left(n+1\right)},$$

(19) $${\hat{G}}_{7\;1}^{\left(n+1\right)}={\hat{y}}_{t}{\hat{G}}_{2\;1}^{\left(n+1\right)}+\left({\hat{x}}_{t}+{\hat{y}}_{t}\right){\hat{G}}_{7\;1}^{\left(n+1\right)}.$$

Solving Equations (15)–(19) for the corresponding Green functions gives

(20) $${\hat{X}}_{t}={\hat{x}}_{t}-\frac{3{\hat{y}}_{t}^{2}\left({\hat{x}}_{t}^{2}-{\hat{x}}_{t}+{\hat{x}}_{t}{\hat{y}}_{t}-2{\hat{y}}_{t}^{2}\right)}{\left(1-{\hat{x}}_{t}-2{\hat{y}}_{t}\right)\left(1-{\hat{x}}_{t}^{2}+{\hat{y}}_{t}-{\hat{x}}_{t}{\hat{y}}_{t}+2{\hat{y}}_{t}^{2}\right)},$$

(21) $${\hat{Y}}_{t}=\frac{{\hat{y}}_{t}^{2}\left(1-{\hat{x}}_{t}+\hat{y}\right)}{\left(1-{\hat{x}}_{t}-2{\hat{y}}_{t}\right)\left(1+{\hat{y}}_{t}-\left({\hat{x}}_{t}-{\hat{y}}_{t}\right)\left({\hat{x}}_{t}+2{\hat{y}}_{t}\right)\right)}.$$

To account for boundary conditions, Equation (3) is utilised, resulting in

(22) $$x_{t}={\hat{x}}_{t}+{\hat{x}}_{t}b_{1}x_{t}+3{\hat{y}}_{t}b_{2}y_{t},$$

(23) $$y_{t}={\hat{y}}_{t}+{\hat{y}}_{t}b_{1}x_{t}+{\hat{x}}_{t}b_{2}y_{t}+2{\hat{y}}_{t}b_{2}y_{t},$$

(24) $$z_{t}={\hat{x}}_{t}+{\hat{y}}_{t}b_{1}y_{t}+{\hat{x}}_{t}b_{2}z_{t}+2{\hat{y}}_{t}b_{2}w_{t},$$

(25) $$w_{t}={\hat{y}}_{t}+{\hat{y}}_{t}b_{1}y_{t}+{\hat{x}}_{t}b_{2}w_{t}+{\hat{y}}_{t}b_{2}w_{t}+{\hat{y}}_{t}b_{2}y_{z}.$$

Solving these equations simultaneously results in

(26) $$x_{t}=\frac{{\hat{x}}_{t}-b_{2}\left({\hat{x}}_{t}-{\hat{y}}_{t}\right)\left({\hat{x}}_{t}+3{\hat{y}}_{t}\right)}{\Delta_{t}},$$

(27) $$y_{t}=\frac{{\hat{y}}_{t}}{\Delta_{t}},$$

(28) $$z_{t}=\frac{\left(1-{\hat{x}}_{t}b_{1}\right)\left(1-b_{2}\left({\hat{x}}_{t}-{\hat{y}}_{t}\right)\right)-\Delta_{t}\left(1-3b_{2}\left({\hat{x}}_{t}-{\hat{y}}_{t}\right)\right)}{3b_{2}\left(1-b_{2}\left({\hat{x}}_{t}-{\hat{y}}_{t}\right)\right)\Delta_{t}},$$

(29) $$w_{t}=\frac{\left(1-b_{1}\left({\hat{x}}_{t}-{\hat{y}}_{t}\right)\right){\hat{y}}_{t}}{1-\left(b_{2}\left({\hat{x}}_{t}-{\hat{y}}_{t}\right)\right)\Delta_{t}},$$

where $b_{1}=B_{1\;1}^{\left(n\right)}$, $b_{2}=B_{6\;6}^{\left(n\right)}=B_{11\;11}^{\left(n\right)}=B_{16\;16}^{\left(n\right)}$, $x_{t}=G_{1\;1}^{\left(n\right)}$, $y_{t}=G_{1\;6}^{\left(n\right)}$, $z_{t}=G_{6\;6}^{\left(n\right)}$, $w_{t}=G_{6\;16}^{\left(n\right)}$ and $\Delta_{t}=\left({\hat{x}}_{t}b_{1}-1\right)\left({\hat{x}}_{t}b_{2}-1\right)+2b_{2}\left({\hat{x}}_{t}b_{1}-1\right){\hat{y}}_{t}-3{\hat{y}}_{t}^{2}b_{1}b_{2}$.

The pivotal elements calculated in this section can now be utilised to derive the relevant operating sensor characteristic. This is presented in the following section.

## 5. Derivation of the Reception Force Response (RFR)

The model for the Sierpinski tetrix inspired sensor will have mechanical loads at the output vertices and an electrical load at the input vertex. The configuration of the proposed device follows a similar arrangement as the Sierpinski gasket fractal-inspired transducer [6]. The sensor generates an electric charge when its front face is subjected to external loads, that is, when the output vertices *B*, *C* and *D* (which correspond to vertex nodes 6, 11 and 16) receive an external load. Expressions for the displacement in the load and backing material are given as

(30) $${\bar{u}}_{L}=A_{L}\exp\left(\frac{-pv_{T}x_{L}}{\Delta xv_{L}}\right)\;\mathrm{and}\;{\bar{u}}_{B}=A_{B}\exp\left(\frac{-pv_{T}x_{B}}{\Delta xv_{B}}\right),$$

where the subscripts *L* and *B* refer to the mechanical load and backing layer, respectively, $v_{T}$ is the wave velocity in the piezoelectric material, $A_{L}$ and $A_{B}$ are constants that represent the forward traveling waves and *p* is the Laplace transform variable. These expressions for the displacement in the load and backing material were found by obtaining solutions to the governing wave equations. At the sensor boundaries, conditions of continuity of displacement and force are applied. Accounting for symmetries in the Green function matrix and by applying the conditions of continuity of displacement, provides

(31) $$u_{1}=u_{A}=A_{B},$$

(32) $$u_{6}=u_{11}=u_{16}=u_{B}=u_{C}=u_{D}=A_{L},$$

where the numbered subscripts attached to the displacement *u* refer to the mechanical displacements at the specified vertices, and the subscripted letters are the mechanical displacements at the fictitious vertices. Furthermore, the force on each vertex is given by $F=A_{r}T$ [6], where $A_{r}$ is the cross-sectional area and *T* is the one-dimensional stress variable. Thus, continuity of force results in

(33) $$u_{1}-u_{A}-\frac{hQ}{Y_{T}\xi }=-\frac{Z_{B}}{Z_{T}}pA_{B},$$

(34) $$u_{B}-u_{6}-\frac{hQ}{Y_{T}\xi }=-\frac{Z_{L}}{Z_{T}}pA_{L},$$

where $h=e_{33}/\epsilon_{33}$ is the piezoelectric constant, *Q* is the electrical charge, $Z_{B}$, $Z_{L}$ and $Z_{T}$ are the mechanical impedances of the backing layer, load and sensor, respectively, and *ξ* is the ratio of the cross-sectional area of each edge to its length. The elements of the boundary condition matrix $B^{\left(n\right)}$ and the vector ${\underline{c}}^{\left(n\right)}$, introduced in Equation (14), are found by utilising Equations (31)–(34) as in a similar manner to [6]. Thus, these are given by

(35) $$B_{ij}=\left\{\begin{array}{cc} \frac{1}{1-p\frac{Z_{B}}{Z_{T}}} & \mathrm{if}\;i=j=1\\ \frac{1}{1-p\frac{Z_{L}}{Z_{T}}} & \mathrm{if}\;i=j=6,\;11\;\mathrm{or}\;16\\ \;\;\;\;\;0 & \mathrm{otherwise} \end{array}\right.$$

and

(36) $$c_{i}=\left\{\begin{array}{cc} \;\;\;\;\;\;\;\;-\frac{hQ}{Y_{T}\xi }\left(\frac{1}{1-p\frac{Z_{B}}{Z_{T}}}\right) & \mathrm{if}\;i=1\\ \left(\frac{hQ}{Y_{T}\xi }-2pA_{L}\frac{Z_{L}}{Z_{T}}\right)\left(\frac{1}{1-p\frac{Z_{L}}{Z_{T}}}\right) & \mathrm{if}\;i=6,\;11\;\mathrm{or}\;16\\ \;\;\;\;\;\;\;\;\;\;\;\;\;\;\;\;\;\;\;\;\;\;\;\;\;0 & \mathrm{otherwise} \end{array}\right.$$

Equations (35) and (36) will be used to determine the expression for the RFR for the Sierpinski tetrix inspired sensor.

**Figure 4:**
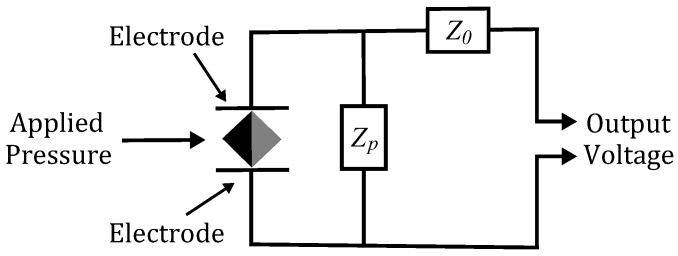
Possible arrangement of the sensor with series $Z_{0}$ and parallel $Z_{p}$ electrical loads.

The RFR is defined as the ratio of the output voltage to the force at the front face of the sensor. The expression for amplitude of the forward propagating wave is given by

(37) $$A_{L}=-\frac{Fv_{L}}{pY_{L}\xi v_{T}},$$

where *F* is the force applied to the front face of the transducer (i.e., to ficticious nodes *B*, *C* and *D*) and $Y_{L}$ is the Young’s modulus of the load. Again, since the pre-fractal sensor device is positioned in a similar arrangement to the Sierpinski gasket fractal-inspired transducer [6], it follows that the expression for the non-dimensionalized RFR is calculated in a similar way. Hence, the non-dimensionalized RFR is

(38) $$\psi_{t}=\frac{VhC_{0}}{F}=\frac{2h^{2}C_{0}\alpha_{t}}{Y_{T}\xi \left(1-\frac{aZ_{T}}{pC_{0}Y_{T}\xi \left(Z_{E,t}+b\right)}\left(1+\frac{h^{2}C_{0}\left(\alpha_{t}+\beta_{t}\right)}{Y_{T}\xi }\right)\right)},$$

where $C_{0}$ is the capacitance, $Z_{E,t}$ is the electrical impedance, $a=Z_{p}/\left(Z_{0}+Z_{p}\right)$, $b=Z_{0}Z_{p}/\left(Z_{0}+Z_{p}\right)$, with $Z_{0}$ and $Z_{p}$ corresponding to the series and parallel electrical loads respectively, as seen in Figure 4, and

(39) $$\alpha_{t}=\frac{3y_{t}-w_{t}-z_{t}}{1-p\frac{Z_{L}}{Z_{T}}}\;\mathrm{and}\;\beta_{t}=\frac{y_{t}-x_{t}}{1-p\frac{Z_{B}}{Z_{T}}}.$$

With the relevant operating characteristic now derived, we can now determine the possible benefits of a sensor based on the design of the Sierpinski tetrix.

## 6. Results

Current manufacturing limitations do not allow for the construction of a piezoelectric Sierpinski tetrix at high generation levels at the dimensions required for an operational frequency in the sub-megahertz range. This is a result of the increasing complexity in the design as the generation level is increased (of course, the scale of the prototype could be increased, in order to attain higher generation levels, but the corresponding operating frequency range would be significantly reduced). Therefore, in this section, a mathematical computer model is employed to test the performance of a Sierpinski tetrix fractal-inspired sensor at generation levels one to five. This is to account for manufacturing restrictions that would be present in higher fractal generation level devices. Furthermore, a comparison of the RFR between the Sierpinski tetrix and gasket devices, at fractal generation level five, and the currently used Euclidean device is presented. The three devices have been modelled on a lead zirconate titanate (PZT-5H) ceramic with the material parameters shown in Table 1. The Sierpinski gasket was used for comparison due to it belonging to the same family of fractal lattices. Specifically, the Sierpinski gasket belongs to the S(3) lattice family and the Sierpinski tetrix is part of the S(4) family [16,26]; where the 3 and 4 indicate the coordination number of the fractal lattices.

The RFR for the Sierpinski tetrix pre-fractal sensors was evaluated by determining the maximum amplitude (*a*), bandwidth (bw) and the gain bandwidth product (gbp), which is the product of the maximum amplitude and bandwidth. The sensor detects ultrasonic energy and then converts it into an electrical signal. The higher the amplitude of the ultrasonic wave, the greater the voltage signal [27]. The bandwidth of the device is often used as the cut-off frequency since this gives the range of frequencies over which the sensor operates efficiently. Previous research has assessed the effectiveness of a transducer by determining its gain bandwidth product [28,29]. This figure of merit is beneficial as it can provide an estimate for the range of frequencies around a particular centre frequency that attains a particular amplitude [30].

### 6.1. Sensor Performance at Varying Fractal Generation Levels

**Figure 5:**
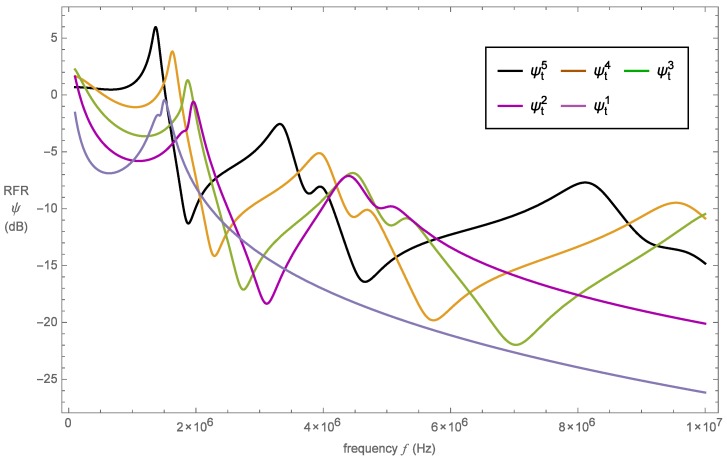
Non-dimensionalized reception force response (RFR) *ψ* (dB) versus frequency *f* (Hz) for the Sierpinski tetrix fractal-inspired sensor at fractal generation levels one to five.

The RFR profiles for the proposed device based on the initial five fractal generations of the Sierpinski tetrix pre-fractals are shown in Figure 5. It is evident from this plot that an increase in the fractal generation level from one to five results in higher maximum amplitudes. Furthermore, it can be seen that fractal generation levels three, four and five produce RFR profiles with more resonances than their generation levels one and two counterparts. In fact, by increasing the fractal generation level beyond level five, we find that the RFR profiles show evidence of more resonances, but this is offset with a reduced maximum amplitude.

**Figure 6:**
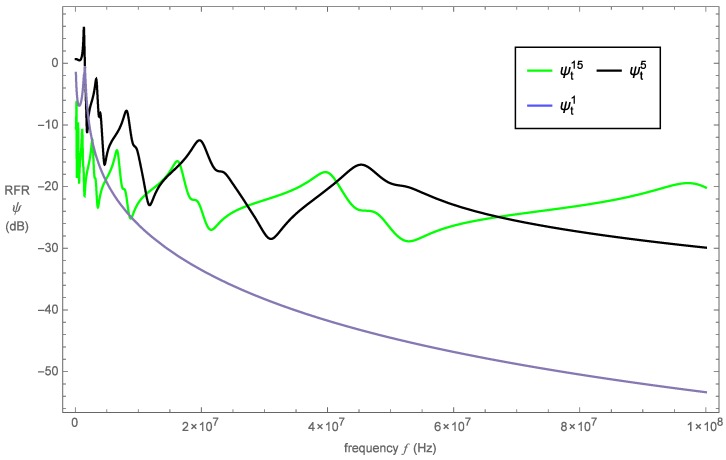
Non-dimensionalized RFR *ψ* (dB) versus frequency *f* (Hz) for the Sierpinski tetrix fractal-inspired sensor at fractal generation levels one, five and fifteen.

The RFR profiles for the proposed device at generation levels one, five and fifteen are shown in Figure 6. As is evident from this plot, the initial fractal generation level has far fewer resonances in comparison to other two devices. This indicates that the device at this generation level has only a single length scale and hence the presence of a single resonance.

The higher the fractal generation level, the greater the presence of resonances, and this is attributed to increased complexity in the pre-fractal design; to be precise, there is a greater range of length scales. However, moving beyond a certain fractal generation level results in a compromise between increased resonances and maximum amplitude. This can be observed when comparing generation level five to fifteen. As the generation level is increased, the size of the length scales decreases, and we find that, for very small length scales, the performance of the sensor at higher frequencies is improved. This is expected since the designs of higher generation levels span a greater range of length scales.

The bandwidth for fractal generation levels one to five is calculated using the same amplitude. That is, the peak amplitude is taken at the resonant frequency of fractal generation level two, since this generation level had the lowest maximum amplitude. In this respect, the bandwidth is likely to increase with the fractal generation. To corroborate this assumption, the three metrics, for each fractal generation level, have been calculated and are tabulated in Table 2.

Additional resonances in the RFR profiles are desired as they increase the operational bandwidth of the device. Single length scale devices can only operate at a single frequency, whilst designs with a range of length scales have the potential to operate over a wide range of frequencies. Thus, to raise the entire RFR output, a device that presents multiple resonances is preferred. The desirable resonant behaviour of the sensor is clearly achieved by increasing the fractal generation level. Furthermore, these resonances occur at lower frequencies. The added advantage of higher fractal generation level devices is the effect it has on the RFR bandwidth. In certain instances, increasing the fractal generation level improves the operational bandwidth at the resonant frequency significantly. For example, due to the additional resonances of the fifth generation level design, the RFR outperforms the first generation device at almost every possible frequency. Additionally, the value of the gain bandwidth product is much more encouraging, as there is a noticeable increase in this figure of merit as the fractal generation level is increased. In some instances, this figure of merit increased more than three-fold over the previous fractal generation level. The gain bandwidth product was calculated using gbp=a×bw, where the raw value for the maximum amplitude (*a*) for each fractal generation level was used before its conversion into decibels.

### 6.2. Comparison between Standard and Pre-Fractal Devices

**Figure 7:**
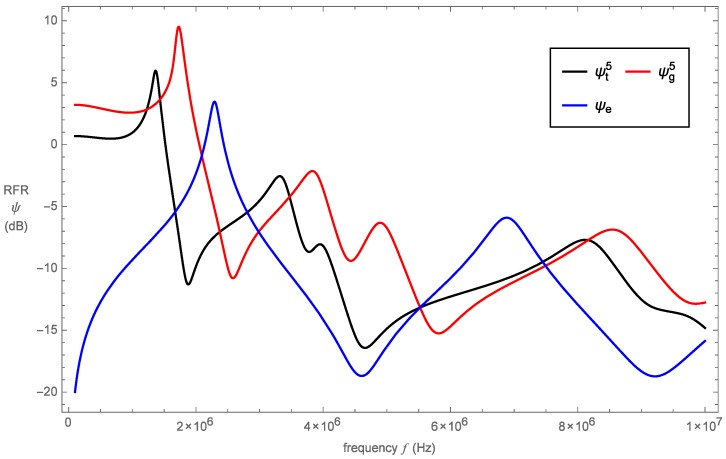
Non-dimensionalized RFR *ψ* (dB) versus frequency *f* (Hz) for the Sierpinski tetrix and Sierpinski gasket fractal-inspired sensors at fractal generation five and the traditional Euclidean sensor.

Comparisons of RFR are illustrated in Figure 7 for the standard and pre-fractal devices. While the sensor designs vary in their construction, for a worthwhile comparison, care has been taken with regard to the construction variables to ensure that the designs have their first resonance around the same frequency. We note that, as expected due to the range of length scales present, the pre-fractal designs demonstrate more resonances than the Euclidean transducer and hence are effective over a wider frequency range. The profiles of the pre-fractal devices are qualitatively similar with the Sierpinski tetrix inspired-device displaying lower amplitudes than the Sierpinski gasket inspired-device at all frequencies. The lower amplitudes present in the tetrix device could suggest that there is a loss in signal as a result of the additional path the ultrasonic wave has to travel. The amplitude of the wave diminishes the further it travels through the lattice structure and thus results in greater attenuation. However, the tetrix device does resonate at lower frequencies than the gasket device and therefore may allow for greater penetration depth.

It was expected that the pre-fractal Sierpinski tetrix sensor would exhibit more resonances than the Sierpinski gasket pre-fractal device on account of its lattice structure covering more length scales. However, the results do not validate this initial assumption, since, in Figure 7, it is observed that the gasket device is equally as resonant. This may relate to the simplification of the tetrix model. The reduction of the three-dimensional model to the one-dimensional mode only accounts for wave propagation in one direction. Similarly, the model of the Sierpinski gasket device restricts attention to wave propagation in the same direction [6]. Performing three-dimensional wave analysis on the Sierpinski tetrix device may yield better sensor performance characteristics in comparison to the Sierpinski gasket device. This analysis is proposed as future work in Section 8. In regards to the maximum amplitude at generation level 5, the tetrix device has $a_{t}^{5}=5.967$ dB and the gasket device has $a_{g}^{5}=9.520$ dB. These amplitudes suggest that the increase in coordination number from 3, for the gasket lattice, to 4, for the tetrix lattice, has reduced the maximum amplitude by 37%. The RFR bandwidth for the Sierpinski tetrix device is $bw_{t}^{5}=0.846$ MHz, while bandwidth of the Sierpinski gasket device is $bw_{g}^{5}=2.022$ MHz. Additionally, the gain bandwidth product for the tetrix device was calculated as $gbp_{t}^{5}=3.341$, which is a decrease of 72% compared to the gasket device, where $gbp_{g}^{5}=11.881$.

Comparisons between the Sierpinski tetrix fractal-inspired sensor and Euclidean sensor are much more encouraging. Figure 7 shows that the pre-fractal device contains more resonances than the traditional design. This is to be expected since the Sierpinski tetrix device benefits from range of length scales while standard designs generally only have a single length scale. The maximum amplitude for the standard device is $a_{e}=3.462$ dB, and thus the Sierpinski tetrix device outperforms the standard device by 72% in terms of this metric. Moreover, the RFR bandwidth surpasses standard designs by an additional 0.583 MHz. As a result of the increased amplitudes and bandwidth exhibited in the Sierpinski tetrix device, the gain bandwidth product is also enhanced; see Table 3. Noting Table 2 and Table 3, all fractal generation levels surpass the standard device in terms of bandwidth. Here, in order to get a fair comparison, the bandwidths for the pre-fractal devices have all been calculated using the 3 dB amplitude of the second generation level. The advantage of pre-fractal designs over Euclidean designs is the presence of more resonances. It has been observed that the initial three fractal generation levels are not as effective in at least one of the figures of merit as the standard device. However, as the generation level is increased, so does the presence of resonances, thus increasing the values of all figures of merit. It was found that higher fractal generation level devices exhibited additional resonances at higher frequencies, while such resonances were absent in lower generation levels. Thus, it may be established that devices designed on high fractal generation levels will most closely resemble those found in nature, for which these systems are far more efficient in operating over a wider range of frequencies, giving rise to improved bandwidths. Therefore, the improvement in these values as the generation level is increased demonstrates that multiple resonances enhance sensor performance. The amplitude at fractal generation level four exceeds the standard device, and fractal generation level three shows a significant improvement in regards to the gain bandwidth product. In particular, there is over a two fold increase at this fractal generation level over that of the standard device. Thus, the results in comparison to a standard design suggest strongly that it would be worthwhile for a prototype based on the Sierpinski tetrix to be built, in order to determine whether experimental results support these theoretical results, especially for fractal generation level five. Three-dimensional printing of electronic sensors and additively-manufactured piezoelectric devices are still emerging technologies [31,32,33]. However, the scope exists for tackling the construction of these three-dimensional piezoelectric structures.

The effect of increasing the fractal generation level resulted with an improvement in all three metrics with the exception of generation level two. In addition, there was a decrease in regards to the operational bandwidth and gain bandwidth product when increasing the fractal generation level from four to five. However, there is concern over the Sierpinski tetrix device performance in comparison to the Sierpinski gasket device at the same fractal generation level. On the other hand, the tetrix device performs much more effectively than currently favoured designs.

### 6.3. Convergence of the Model

The convergence of the fractal generation level for the RFR is presented in this section. This was achieved by computing the absolute value of the difference between successive fractal generations for up to 50 generation levels. That is,

(40) $$\gamma_{r}=\frac{{∥\psi \left(f;n\right)-\psi \left(f;n+1\right)∥}_{2}}{\max\psi (f;n)},$$

where ψ(f;n) is the RFR at frequency *f* and generation level *n*. Previous research [6,11] has also applied this same technique in order to determine the point of convergence for the Sierpinski gasket fractal-inspired transducer.

**Figure 8:**
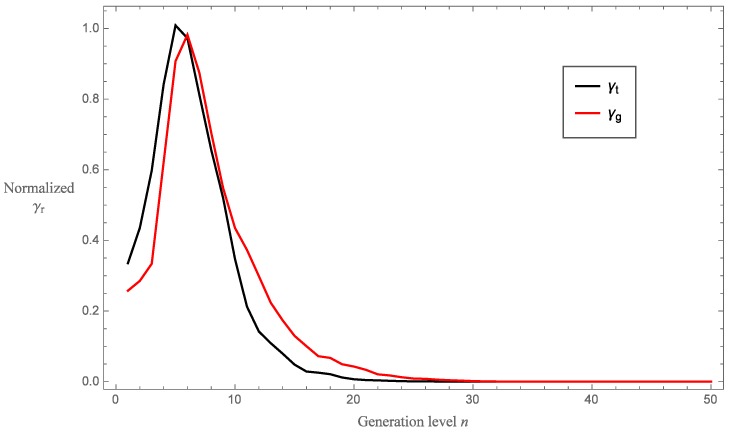
Normalized RFR for the Sierpinski tetrix ($\gamma_{t}$) and Sierpinski gasket ($\gamma_{g}$) devices as a function of fractal generation level *n*.

Figure 8 illustrates the points of convergence of the RFR for the Sierpinski tetrix and Sierpinski gasket fractal-inspired sensors, and has been normalized between zero and one. We see that significant improvements can be made on the RFR by increasing the generation level from one to five. Thereafter, the differences between each successive generation level decreases before converging. The Sierpinski tetrix device converges at a lower fractal generation level when compared with the gasket device. Using a 5% tolerance level, the Sierpinski tetrix device converges by fractal generation level n=25, while the Sierpinski gasket device converges by fractal generation level n=32. At the 1% tolerance level, the convergences are n=28 and n=34 for the Sierpinski tetrix and gasket devices, respectively. From a manufacturing perspective, this is positive as it shows that a device which incorporates a pre-fractal with a high generation level is not required. The trade-off between design intricacy and device performance is maximised at around generation level five, which is possible with the current prototyping technology.

## 7. Discussion

This paper investigated the RFR of a Sierpinski tetrix fractal-inspired transducer for the use of sensor applications. The Green function renormalization technique was utilised as a method for obtaining the sensor operating characteristic and was modelled on a PZT-5H ceramic. The Sierpinski tetrix fractal-like sensor was examined by constructing the lattice counterparts, a method previously performed for the Sierpinski gasket [6,11] and Sierpinski carpet devices [25], to derive and analyse the RFR.

As a result of current manufacturing procedures, the construction of pre-fractal sensors would be restricted to low fractal generation levels. This is a consequence of the reduction in the size of the length scales as the generation level is increased. In this paper, the first five fractal generation levels have been modelled. Additionally, fractal generation level fifteen has been included to determine what effect higher fractal generation levels have on the performance of the sensor. The point of convergence of the sensors performance was also studied. From this analysis, it was shown that the RFR converged by fractal generation level n=25 with a tolerance level of 5% and converged at n=28 with a 1% tolerance level.

Computer models are a valuable resource to predict the performance of hypothetical designs as they help minimise the associated time and costs involved in manufacturing new ultrasonic sensors. Additionally, they assist in determining the most effective configuration of new designs. Thus, to assess the performance of the Sierpinski tetrix fractal-inspired sensor, a computer model was utilised to plot the RFR as a function of the operating frequency. This was then compared to a previously investigated Sierpinski gasket fractal-inspired transducer, in reception mode as well as the conventional 1–3 composite sensor. As expected, the tetrix device experienced more resonances at a wider range of frequencies than the standard sensor. Furthermore, an increase in the fractal generation level led to a more resonant device with the presence of higher frequency resonances. Thus, fractal-inspired sensors demonstrate suitability for a various number of purposes. For the Sierpinski tetrix pre-fractal, an increase in fractal generation level was mostly followed by an increase in the device’s RFR amplitude, bandwidth and gain bandwidth product. For fractal generation level five, these figures of merit were all reduced in comparison to the previously investigated Sierpinski gasket-inspired sensor. However, in regards to device performance over traditional designs, the analysis presented in this paper indicates a substantial increase in each of these figure of merits. These results do therefore suggest the possibility that a Sierpinski tetrix fractal-inspired sensor could be suitable in ultrasonic design to enhance current transducer performance in reception mode.

## 8. Conclusions

This paper investigated the theoretical performance of a Sierpinski tetrix fractal-inspired sensor by utilising the Green function renormalization method. Given the simplified nature of the model, it could be beneficial to investigate this structure using finite element analysis akin to that done on the Sierpinski gasket pre-fractal in [11,24]. Utilising this method would allow for the reintroduction of longitudinal and shear propagating waves into the model. Sensor performance optimization could also be employed with the aim of improving the values of the RFR bandwidth, amplitude and gain bandwidth product. This could be achieved by sampling different material parameters, such as piezoelectric constant and Young’s modulus [34]. This provides an opportunity to ascertain a range of materials that could be used in the creation of novel fractal-inspired ultrasonic sensors.

## Figures and Tables

**Table 1:** Sensor material parameters for a PZT-5H ceramic [15].

	Symbol	Magnitude	Dimensions
Elastic constant	$c_{33}$	$11.74\times 10^{10}$	N$\cdot m^{-2}$
Piezoelectric stress coefficient	$e_{33}$	23.3	C$\cdot m^{-2}$
Permittivity tensor element	$\epsilon_{33}$	$1.47\times 10^{3}$	-
Density	$\rho_{T}$	$7.5\times 10^{3}$	kg$\cdot m^{-3}$
Parallel electrical load	$Z_{p}$	$10^{3}$	Ω
Series electrical load	$Z_{0}$	50	Ω
Fractal length	*l*	1	mm

**Table 2:** Figures of merit for the Sierpinski tetrix pre-fractal sensors at generation levels one to five, where the bandwidth is calculated using the amplitude at the resonant frequency for fractal generation level one.

Generation (*n*)	Amplitude (*a*) dB	Bandwidth (*bw*) MHz	Gain Bandwidth Product (*gbp*) MHz
1	−0.448	0.415	0.375
2	−0.597	0.386	0.336
3	1.296	0.941	1.269
4	3.825	1.765	4.259
5	5.967	1.521	6.010

**Table 3:** Figure of merits for the Euclidean, Sierpinski gasket and tetrix pre-fractal sensors at generation level five. The bandwidth of all devices has been calculated using the amplitude at the resonant frequency of the Euclidean sensor.

	Amplitude (*a*) dB	Bandwidth (*bw*) MHz	Gain Bandwidth Product (*gbp*) MHz
Sierpinski Tetrix	5.967	0.846	3.341
Sierpinski Gasket	9.520	2.022	11.811
Euclidean	3.462	0.263	0.584
